# The Neural Correlates of Effortful Cognitive Processing Deficits in Schizophrenia: An ERP Study

**DOI:** 10.3389/fnhum.2021.664008

**Published:** 2021-05-28

**Authors:** Chen-Guang Jiang, Jun Wang, Xiao-Hong Liu, Yan-Ling Xue, Zhen-He Zhou

**Affiliations:** Department of Psychiatry, The Affiliated Wuxi Mental Health Center of Nanjing Medical University, Wuxi, China

**Keywords:** schizophrenia, the face-vignette task, effortful cognitive processing, event-related potential, frontal cortex site, prefrontal cortex site

## Abstract

**Background:** Individuals’ information processing includes automatic and effortful processes and the latter require sustained concentration or attention and larger amounts of cognitive “capacity.” Event-related potentials (ERPs) reflect all neural activities that are related to a certain stimulus. Investigating ERP characteristics of effortful cognitive processing in people with schizophrenia would be helpful in further understanding the neural mechanism of schizophrenia.

**Methods:** Both schizophrenia patients (SCZ, *n* = 33) and health controls (HC, *n* = 33) completed ERP measurements during the performance of the basic facial emotion identification test (BFEIT) and the face-vignette task (FVT). Data of ERP components (N100, P200, and N250), BFEIT and FVT performances were analyzed.

**Results:** Schizophrenia patients’ accuracies of face emotion detection in the BFEIT and vignette emotion detection in the FVT were both significantly worse than the performance of the HC group. Repeated-measures ANOVAs performed on mean amplitudes and latencies revealed that the interaction effect for group × experiment × site (prefrontal, frontal, central, parietal, and occipital site) was significant for N250 amplitude. In FVT experiment, N250 amplitudes at prefrontal and frontal sites in schizophrenia group were larger than those of HC group; the maximum N250 amplitude was present at the prefrontal site in both the groups. For N250 latency, the interaction effect for group × experiment was significant; N250 latencies in the schizophrenia group were longer than those of the HC group.

**Conclusion:** Schizophrenia patients present effortful cognitive processing dysfunctions which reflect in abnormal ERP components, especially N250 at prefrontal cortex and frontal cortex sites. These findings have important implications for further clarifying the neural mechanism of effortful cognitive processing deficits in schizophrenia.

## Introduction

Schizophrenia protect is a severe mental disorder characterized by destruction of thinking, sense of self, emotional response, logical reasoning, cognition, perceptions and volitional behavior. Among all the major symptoms of schizophrenia, cognitive dysfunction authentically contributes to the disabling nature of the disorder (Bowie et al., 2006; Stern, 2012). Systematic studies have confirmed that cognition is an individual’s process of obtaining and applying knowledge, that is, a process of processing information (Tomasello and Call, 2011). Individuals’ information processing includes automatic processes and effortful processes. Automatic cognition processing requires almost no attention to be allocated to the task at hand and in many instances is executed in response to a specific stimulus. Correspondingly, effortful cognition, namely as effortful information processing, depends on attentional capacity and usually can be tested by an “effort-demanding” cognitive task (i.e., tasks that require sustained concentration or attention or require larger amounts of cognitive “capacity”) (Tancer et al., 1990; Hammar and Ardal, 2012; Rodriguez-Larios et al., 2020).

The vulnerability model of schizophrenia was derived from the integration of heredity, abnormal brain structure, impaired brain functioning, physiological and psychological development and early learning (Yank et al., 1993). Reduced availability of processing resources is the critical feature of the vulnerability model of schizophrenia (Rund and Landrø, 1990). Studies have indicated that schizophrenia patients also experience severe deficiencies in resources availability for effortful controlled processing (i.e., effortful cognitive dysfunction) (Granholm et al., 2007; Patrick et al., 2015). Previously a study investigated the relationship between early visual information processing deficits and effortful processing resource allocation (Granholm et al., 2007). In this study, both chronic schizophrenia patients and healthy controls (HCs) performed a span of apprehensive task, and pupillary responses were recorded as an index of resource allocation or mental effort during the task. Their results revealed that patients displayed reduced effortful resource allocation and impaired detection accuracy. Another study compared the effortful decision making of schizophrenia patients versus HCs by focusing on the effort expended when completing a rewards task; the findings also revealed a pattern of inefficient effortful decision making in schizophrenia patients relative to HCs (McCarthy et al., 2016).

Effortful cognitive processing was involved in both social cognition and neurocognition. For example, effortful emotional cognitive processing is a kind of effortful cognition that may supply top-down as well as goal-directed reappraisal of emotion-laden stimuli to contextually modulate affect-driven responding (Phillips et al., 2003; Ochsner and Gross, 2005). The essential characteristic of effortful emotional cognition is attributable to either social cognition or neurocognition. Effortful emotional cognition can be measured by a face-vignette task (FVT), which is an information-processing task with a high processing load, and individuals’ performances on FVT represent their available processing resources (Patrick et al., 2015). Previous studies have confirmed that patients with schizophrenia exhibit impairments in effortful emotional processing (Rowland et al., 2012; Patrick et al., 2015). Cognitive deficits, one of the major symptom dimensions occurring early in the stage of schizophrenia and relatively stable over time, have been considered as a potential treatment target (Rowland et al., 2012; MacKenzie et al., 2018).

Event-related potentials (ERPs), based on electroencephalogram (EEG) recordings, are electrophysiological responses reflecting cognition-related neural activities that can be evoked by certain stimuli during an experiment. ERPs have become a prominent approach for studying the neural mechanisms involved in cognitive function. To date, although previous studies have suggested that schizophrenia patients have impaired effortful cognitive processing, the ERP characteristics of effortful cognitive processing in schizophrenia have not been reported yet, although it would be of help to understand the neural mechanism of this disease. Furthermore, clarifying such ERP characteristics would have implications for better understanding the etiology, clinical features and treatment strategies in schizophrenia.

On the basis of the foregoing, it is supposed that people with schizophrenia would have abnormal ERP responses due to effortful emotional processing deficits. To test this hypothesis and investigate the neural mechanism of effortful cognitive processing in schizophrenia, both schizophrenia patients and HCs performed a Basic facial emotion identification test (BFEIT) and a FVT, to reflect their automatic cognitive function and effortful cognitive function, respectively. ERPs evoked by BFEIT and FVT were synchronously measured.

## Materials and Methods

### Time and Setting

This study was conducted from January 01, 2017, to July 31, 2020 in the affiliated Wuxi Mental Health Center of Nanjing Medical University. The study protocol was approved by the Ethics Committee of Wuxi Mental Health Center and was conducted in accordance with the Declaration of Helsinki (Reference No. 2017LLKY007).

### Participants

All participants with schizophrenia were recruited from inpatients of Wuxi Mental Health Center. The inclusion criteria were: (a) met the criteria of schizophrenia according to the Diagnostic and Statistical Manual of Mental Disorders, Fifth edition (DSM-5), (b) Chinese Han, aged 18 to 65 years, (c) had no current or history of neurological illness or any other kind of severe physical illness that would affect his/her cognitive function, (d) educational level no less than junior middle school (normally 9 years), (e) volunteer to participate in this study, and (f) clinical stable enough to fulfill this study. The exclusion criteria were: (a) met criteria of any other mental disorder according to DSM-5, (b) treated by electroconvulsive therapy (ECT) or modified ECT within 6 months before recruitment, (c) had nicotine/other substance misuse or dependence within the latest 6 months, and (d) visual impairment that cannot be corrected to satisfy the demand of the current study. Healthy controls were recruited from local citizen through advertisement. The inclusion criteria were: (a) met no criteria of any kind of mental disorder according to DSM-5, and (b) to (e) criteria as the schizophrenia patients group. The exclusion criteria of HCs were the same as (c) and (d) items as the patients group.

All participants provided written informed consent. Considering that some schizophrenia patients might have impaired capacity to provide consent even if they are clinically stable, we also got consent from patients’ guardians or close relatives. All participants were compensated 300.00 Chinese Yuan (equals to about 45 US dollars) for their time taking part in this study.

### Measurements

#### Basic Facial Emotion Identification Test

**FIGURE 1:**
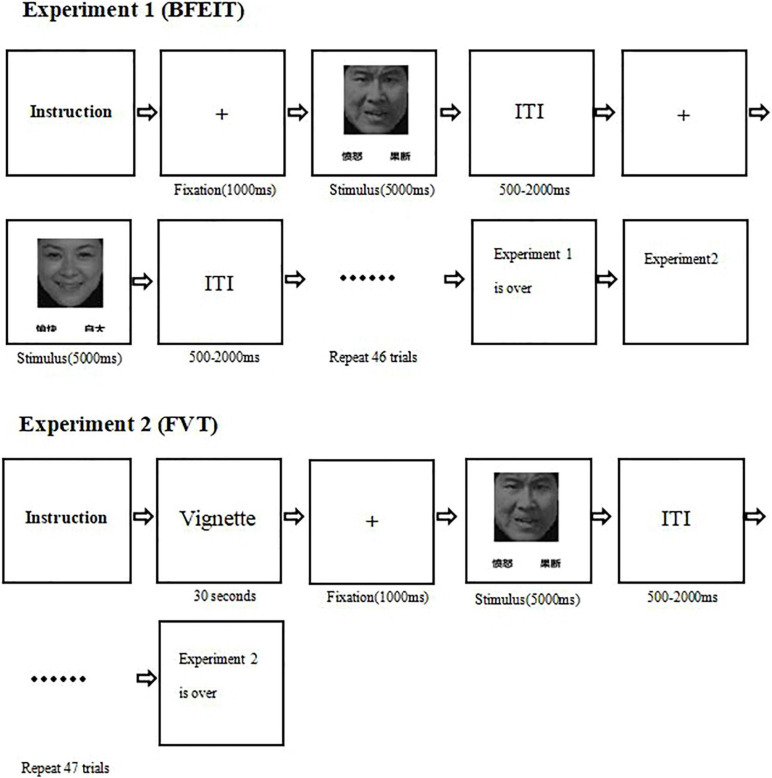
A diagram illustrating the experimental procedures (experiment 1: the measurement of the basic facial emotion identification test; experiment 2: the measurement of the face-vignette task). 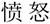, angry; 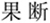: determined; 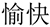, happy; 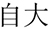, smugness. ITI, inter-trial interval.

In the BFEIT task, there were six categories of basic emotions (i.e., happy, angry, sad, fear, surprise, and disgust) which were selected from the Chinese facial affective picture system (Gong et al., 2011). All pictures were black-and-white photographs with hair removed. Each category of facial emotion types included 8 pictures and the number of male and female face pictures were balanced across each emotion category. The procedure of BFEIT is sketched in Figure 1. Briefly, in each trial, following a centrally presented fixation (“ + ”, 1.0 × 1.0 cm, last for 1000 ms), there was a facial picture with two choices below. One choice represented the basic emotion matched to the face picture and the other one conveyed a non-basic emotion (i.e., guilty, smugness, painful, determined, hopeful and insulted). The position (bottom left or bottom right) of the two choices were pseudo-random. Participants were required to make a choice to classify the emotion represented by the picture as quickly and accurately as possible, with a response deadline of 5000 ms, by pressing a labeled keypad. The next trial started following an inter-trial interval (ITI) with a range varied randomly from 500 to 2000 ms.

#### Face-Vignette Task

**FIGURE 2:**
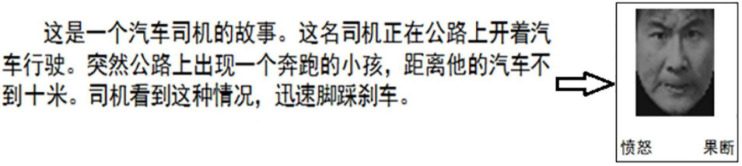
An example of a trial of the FVT. The situational vignette is displayed as follows (in Chinese): This is a story about a bus driver. The driver is driving a bus on the road. Suddenly, he sees a running child on the road no more than 10 m ahead of his bus. He quickly steps on the brakes. Followed by the vignette, there appears a picture of a face on the upper and two choices (angry vs. determined) on the lower position. Subjects are required to choose the better one that reflects the given scenario. 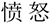, angry; 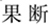: determined.

The procedure of FVT was designed similarly as described in a previous study (Patrick et al., 2015). A succinct situational vignette was constructed, for each face picture, to convey an above mentioned non-basic emotion. Before the test in participants of this study, the intended emotion for each vignette was validated by seven Chinese undergraduates. The mean accuracy was 0.91 [standard deviation (SD) = 0.03], and the observed inter-rater reliability was 0.75. According to the specific emotional category (e.g., a fearful facial expression paired with a painful vignette), the face-vignette pairs were matched such that each vignette was inconsistent with the facial expression (see Figure 2). The specific face-vignette pairs included sadness-guilty, happy-smug, fear-painful, angry-determined, disgusted-insulted and surprised-hopeful. During the FVT trial, the vignette was presented first and the participants were required to read it aloud. After the participants well understood the vignette, he/she could press the “SPACE” of a keyboard to continue. Then a fixation presented in the center of the screen for 1000 ms followed by a face picture. Be different from the BFEIT, participants were required to choose the emotion that best matching the vignette by pressing a labeled keypad (include three items, i.e., face responses, vignette responses and random responses). The procedure of FVT is also sketched in Figure 1.

Both BFEIT and FVT were designed and performed via E-Prime software version 3.0 (Psychology Software Tools Inc, Sharpsburg, PA, United States). All stimuli were presented on a 19-inch computer screen with 1280 × 1024 resolution and a 60 hertz (Hz) refresh rate. Participants were seated in a moderate light and sound attenuated room in front of the screen at a distance of around 60 cm. Participants were asked to complete BFEIT (experiment 1) and then FVT (experiment 2). Before the formal trial, there was a practice procedure (12 trials) to ensure that participants understood the task.

### Electroencephalogram Recordings and ERP Analysis

The BioSemi Active Two system was employed to record EEGs in continuous mode at a 500 Hz digitization rate. According to the international 10/20 system, a customized BrainCap (EasyCap, Herrsching, Germany) containing 64 Ag/AgCl ring-type electrodes was arranged for EEG recordings. Vertical and horizontal electrooculography (EOG) was monitored by additional electrodes placed below and on the external canthi of the left eye. Electrode impedances were below 5 kiloohm (kΩ). The EEG and EOG were filtered by 0.05–100 Hz bandpass filter. The left and right mastoids served as references, and the ground electrodes were placed under the left clavicle site. ERPs were only derived from participants’ correct responses.

The EEG data were processed offline using Brain Vision Analyzer 2.0 (Brain Products GmbH, Munich, Germany) and re-referenced offline to the averaged left and right mastoids. A bandpass filtered between 0.1 and 30 Hz using a zero-phase shift Butterworth filter was used. In each single trial, the EOG was eliminated by the independent component analysis (ICA) algorithm. Data were segmented by stimulus marker from −200 to 1000 ms. Segments were baseline corrected using −200 to 0 ms prestimulus time and were eyeblink corrected using established measures (Monaghan et al., 2019). Artifact rejection for individual channels was performed, and a given segment was rejected if the voltage gradient exceeded 50 microvolts (μV)/ms, the amplitude was ±75 μV, or the signal was flat (<0.5 μV for more than 100 ms). Segments were averaged across stimulus markers. The time-windows locked in each peak were selected.

Grand average ERP responses to target stimuli in experiment 1 and experiment 2 were computed across all participants, and three distinct ERP components, i.e., N100, P200, and N250, were identified and used for statistical analyses based on their distinctive polarities, latencies and topographic maps. In this study, following the stimulus onset, N100 data were measured in a time window between 80 and 150 ms; P200 data were measured in a time window between 150 and 230 ms; and N250 data were measured in a time window between 200 and 350 ms.

According to the scalp topographical distribution of grand-averaged ERP activity in this study, a set of available electrodes was used for statistical analyses. Nineteen electrode sites (AF3, AFz, AF4, F1, F2, Fz, F3, F4, C1, C2, Cz, C3, C4, P1, Pz, P2, PO3, PO4, and POz) were selected and classified into the following five regions of sites: prefrontal site (AF3, AFz, and AF4), frontal site (F1, F2, Fz, F3, and F4), central site (C1, C2, Cz, C3, and C4), parietal site (P1, Pz, and P2), and occipital site (PO3, POz, and PO4). The peak amplitude and corresponding latency of each ERP component and electrode site were measured. And then the average amplitude and latency for each of the five brain regions were calculated.

### Statistical Analysis

All data were analyzed using Statistical Program for Social Sciences software version 22.0 (SPSS, IBM Corp., United States). Quantitative data were compared between the two groups by independent *t* tests (two-tailed) and quantitative data by Pearson chi-square test. The amplitudes and the latencies of ERP components (N100, P200, and N250) in each brain regions (prefrontal, frontal, central, parietal and occipital sites) were compared between the schizophrenia group and the HC group using a 2 (group, schizophrenia vs. HCs) × 2 (experiment, BFEIT vs. FVT) × 5 (brain region, prefrontal vs. frontal vs. central vs. parietal vs. occipital) repeated-measures analysis of variance (ANOVA). Effect sizes were estimated using η*^2^* and Cohen’s *d*. The degrees of freedom of the *F* ratio were corrected using the Greenhouse-Geisser method. Least square difference (LSD) tests were performed as *post hoc* analyses if needed. The Pearson’s correlation analysis was conducted between the amplitudes and latencies of N250 and the PANSS scores respectively. Alpha values of 0.05 were considered significant.

## Results

### Demographic Characteristics of Participants

In line with the inclusion and exclusion criteria, data of 33 schizophrenia patients and 33 HCs were retained for analysis after ruling out those incomplete or low-quality data. The demographic characteristics and clinical information of them are shown in Table 1. There were no significant differences in mean age, educational level, duration of illness, handedness and male-female ratio between the two groups. For patients, the mean lurasidone-equivalent dose was 75.8 ± 4.1 mg/d as calculated according to the previous report (Ng-Mak et al., 2019).

**TABLE 1:** Demographic characteristics and clinical information of participants [mean (SD)].

**Variable**	**Schizophrenia patients (*n* = 33)**	**HCs (*n* = 33)**	**Test statistic**
Age (year)	33.8 (7.7)	32.6 (5.8)	*t* = 0.742, *p* = 0.461
Age range	19–44	22–45	–
Sex (M/F)	20/13	21/12	χ^2^ = 0.064, *p* = 0.800
Education (years)	13.3 (2.8)	14.3 (1.9)	*t* = 1.712, *p* = 0.920
PANSS-Tot scores	63.8 (16.3)	–	–
PANSS-Pos	15.8 (5.8)	–	–
PANSS-Neg	15.1 (5.0)	–	–
PANSS-Gen	32.8 (8.2)	–	–
Handedness (R/M/L)	12/10/11	13/10/10	χ^2^ = 0.195, *p* = 0.901
Duration of illness	11.2 (7.3)	–	–
Medicine(A/C/O/R)	6/10/9/8		

*HC, healthy control; SD, standard deviation; R, right, M, mixed, L, left; PANSS, Positive and Negative Syndrome Scale; Tot, total scores; Pos, positive symptoms factor scores; Neg, negative symptoms factor scores; Gen, general symptoms factor scores. Medicine (A: amisulpride C: clozapine O: olanzapine, R: risperidone).*

### Comparisons of BFEIT and FVT Performance Accuracy Between the Two Groups

For BFEIT, independent sample *t* test results indicated that there was significant difference in emotion identification accuracy between the schizophrenia group (Mean = 69.3%, SD = 11.8%) and the HC group (Mean = 77.1%, SD = 12.4%) (*t* = 2.629, *p* = 0.011). For FVT, significant difference was found in correct vignette response proportions between the schizophrenia patients (face response proportions: Mean = 29.9% SD = 13.8%; vignette response proportions: Mean = 67.9% SD = 14.1%) and the HCs (face response proportions: Mean = 22.6%, SD = 8.9%; vignette response proportions: Mean = 75.3%, SD = 8.9%) (*t* = 2.546, 2.659; *p* = 0.013, 0.008). However, there were no significant differences in random response proportions between the schizophrenia group (Mean = 2.2%, SD = 0.1%) and the HC group (Mean = 2.1%, SD = 0.1%) (*t* = 1.231, *p* = 0.168). The performance of the HC group was much better than that of the schizophrenia group.

### Comparisons of RTs Between the Schizophrenia Group and the HC Group

For BFEIT, there were significant differences in RTs for target stimuli between the schizophrenia group (Mean = 750.3 ms, SD = 146.2 ms) and the HC group (Mean = 609.5 ms, SD = 102.8 ms) (*t* = 4.531, *p* = 0.000). For FVT, similar differences were found between the two groups. The average RTs for target stimuli of schizophrenia patients (Mean = 808.7 ms, SD = 97.7 ms) was much longer than that of the HC group (Mean = 621.1 ms, SD = 66.1 ms) (*t* = 9.138, *p* = 0.000).

### ERP Data Analysis

For BFEIT, the average number of trials for ERP analyzing were 32.34 ± 5.44 in the schizophrenia group and 36.02 ± 5.87 in the HC group. For FVT, the average number of trials for ERP analyzing were 31.75 ± 6.59 in the schizophrenia group and 35.73 ± 4.46 in the HC group.

Using N100, P200 and N250 as dependent variables, a 2 × 2 × 5 repeated-measures ANOVA was performed on mean amplitudes and mean latencies, respectively, with the group (schizophrenia group vs. HC group) as the between-subjects factor and the experiment (experiment 1 vs. experiment 2) and site (prefrontal, frontal, central, parietal, and occipital) as the within-subjects factor.

#### N100 Component

**FIGURE 3:**
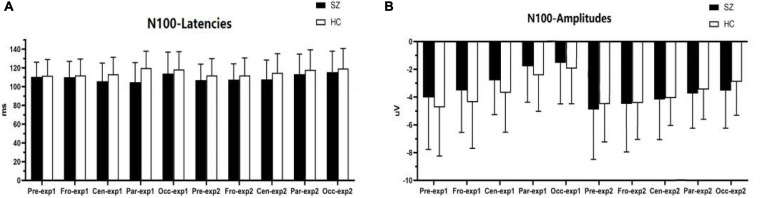
Comparisons of N100 latencies **(A)** and amplitudes **(B)** between the schizophrenia group and HC group within ROIs and experiments. SZ, schizophrenia; HC, healthy control; exp, experiment; Pre, prefrontal, Fro, frontal, Cen, central, Par, parietal, Occ, occipital.

As shown in Figures 3, 6A–D, for N100 amplitude, the three following interaction effects involving the factor “group” failed to reach significance: group × experiment × site (F*_1_, _78_* = 0.025, *p* = 0.914, η*2* = 0.000); group × experiment (F*_1,64_* = 0.025, *p* = 0.174, η*2* = 0.029); and group × site(F*_1,83_* = 0.475, *p* = 0.542, η*2* = 0.007).

For N100 latency, the three following interaction effects involving the factor “group” also failed to reach significance: group × experiment × site (*F_2,135_* = 2.215, *p* = 0.110, η*2* = 0.033); group × experiment (*F_1,64_* = 0.053, *p* = 0.818, η*2* = 0.001); and group × site (*F_2,108_* = 1.113, *p* = 0.324, η*2* = 0.017).

#### P200 Component

**FIGURE 4:**
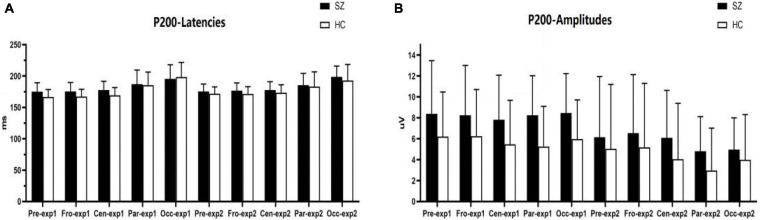
Comparisons of P200 latencies **(A)** and amplitudes **(B)** between the schizophrenia group and HC group within sites and experiments. SZ, schizophrenia; HC, healthy control; exp, experiment; Pre, prefrontal, Fro, frontal, Cen, central, Par, parietal, Occ, occipital.

As shown in Figures 4, 6A–C,E, for P200 amplitudes, the three following interaction effects involving the factor “group” failed to reach significance: group × experiment × site (*F_1,95_* = 0.791, *p* = 0.422, η*2* = 0.012), group × experiment (*F_1,64_* = 0.925, *p* = 0.340, η*2* = 0.014), and group × site (*F_1,82_* = 0.437, *p* = 0.558, η*2* = 0.007).

**FIGURE 5:**
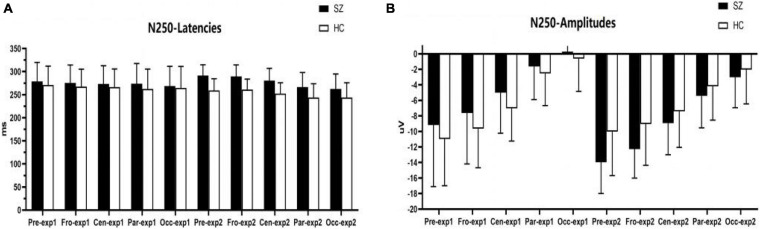
Comparisons of N250 latencies **(A)** and amplitudes **(B)** between the schizophrenia group and HC group within sites and experiments. SZ, schizophrenia; HC, healthy control; exp, experiment; Pre, prefrontal, Fro, frontal, Cen, central, Par, parietal, Occ, occipital.

**FIGURE 6:**
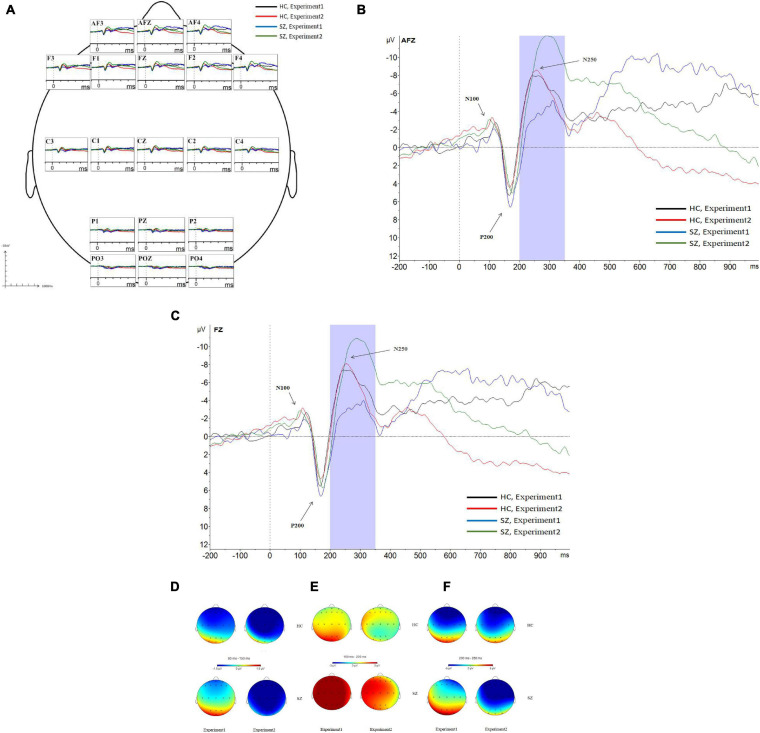
Grand average event-related potentials (ERPs) at the AF3, AFz, AF4, F1, F2, Fz, F3, F4, C1, C2, Cz, C3, C4, P1, Pz, P2, PO3, PO4, and POz sites for all participants during experiment 1 and experiment 2 **(A)**. Following stimulus onset, the N100 component was measured in a time window within 80–150 ms, the P200 component was measured in a time window within 150–230 ms, and the N250 component was measured in a time window within 200–350 ms. In experiment 2, N250 amplitudes at the prefrontal site and frontal site in the SZ group were larger than those of the HC group; N250 latencies in the SZ group were longer than those of the HC group **(B,C)**. Topographic distributions of the N100 components **(D)**, P200 components **(E)**, and N250 components **(F)** in the SZ group and HC group. SZ, schizophrenia; HC, healthy control.

For P200 latency, the three following interaction effects involving the factor “group” failed to reach significance: group × experiment × site (*F_2,128_* = 2.666, *p* = 0.073, η*2* = 0.040), the interaction effect for group × experiment (*F_1,64_* = 0.016, *p* = 0.901, η*2* = 0.000), and group × site was not significant (*F_1,95_* = 0.717, *p* = 0.451, η*2* = 0.011).

#### N250 Component

As shown in Figures 5, 6A–C,F, for N250 amplitudes, the interaction effect for group × experiment × site was significant (*F_1,87_* = 5.992, *p* = 0.009, η*2* = 0.086).

In experiment 1, the interaction effect for group × site was not significant (*F_1,83_* = 0.612, *p* = 0.476, η*2* = 0.009), and the main effect for site was significant (*F_1,83_* = 127.404, *p* = 0.000, η*2* = 0.666); however, the main effect for group was not significant (*F_1,64_* = 1.910, *p* = 0.172, η*2* = 0.029). There were no significant differences in N250 amplitudes between the schizophrenia group and the HC group in experiment 1, and the maximum N250 amplitude was observed in the prefrontal site.

In experiment 2, the interaction effect for group × site was significant (*F_1,83_* = 4.018, *p* = 0.038, η*2* = 0.059). The simple effect for the group within sites was significant (*F_1,64_* = 10.674, *p* = 0.002, η*2* = 0.143). N250 amplitudes at the prefrontal site and frontal site in the schizophrenia group were larger than those of the HC group (*F_1,64_* = 10.647, 8.064; *p* = 0.002, 0.006; η*^2^* = 0.143, 0.112), and no differences were observed in other sites. The simple effect for site within groups was significant (for schizophrenia, *F_3,62_* = 42.867, *p* = 0.000, η*^2^* = 0.675; for HC group, *F_3,62_* = 23.664, *p* = 0.000, η*2* = 0.534). The maximum N250 amplitude was noted at the prefrontal site in both groups.

For N250 latency, the interaction effect for group × experiment × site was not significant (*F_2,114_* = 0.613, *p* = 0.053, η*2* = 0.090), and the interaction effect for group × site was not significant (*F_2,97_* = 0.570, *p* = 0.521, η*2* = 0.009); however, the interaction effect for group × experiment was significant (*F_1,64_* = 4.228, *p* = 0.044, η*2* = 0.062). In experiment 1, the simple effect for the group within sites was not significant (*F_1,64_* = 0.798, *p* = 0.375, η*2* = 0.012). In experiment 2, the simple effect for the group within sites was significant (*F_1,64_* = 23.582, *p* = 0.000, η*2* = 0.269); N250 latencies in the schizophrenia group were longer than those in the HC group.

### Correlation Analysis Between N250 and PANSS Scores

The Pearson’s correlation analysis was conducted between the amplitudes and latencies of N250 at AFz and Fz electrode sites and the PANSS scores, respectively. As shown in Table 2, no significant correlation was found between any two parameters.

**TABLE 2:** Correlations between N250 and PANSS.

	**N250 amplitudes**		**N250 latencies**	
	**AFZ**	**FZ**	**AFZ**	**FZ**
PANSS-Tot	*r* 0.061	0.020	0.199	0.123
	*p* 0.737	0.910	0.267	0.494
PANSS-Pos	*r* 0.193	0.145	0.242	0.196
	*p* 0.281	0.422	0.174	0.275
PANSS-Neg	*r*−0.080	−0.119	−0.038	−0.148
	*p* 0.657	0.508	0.833	0.410
PANSS-Gen	*r* 0.033	0.011	0.247	0.198
	*p* 0.583	0.950	0.165	0.270

*PANSS, Positive and Negative Syndrome Scale; Tot, total scores; Pos, positive symptoms factor scores; Neg, negative symptoms factor scores; Gen, general symptoms factor scores.*

## Discussion

This study is the first to investigate the ERP characteristics of effortful cognitive processing in schizophrenia using a face-vignette task. In this study, the capacity of effortful cognitive processing was determined by the ability of schizophrenia patients to apply contextual information when judging the meaning of facial expressions, while the capacity of automatic cognitive processing was determined by the sample emotion identification task. Our findings showed that the emotion identification accuracy of normal controls was higher than that of the schizophrenia patients in sample emotion identification task; however, schizophrenia patients exhibited poor face-vignette task performance, i.e., the face response proportions of the normal controls were lower than those of the schizophrenia patients, and the vignette response proportions of the normal controls were higher than those of the schizophrenia patients. Most importantly, we found that N250, which was evoked by target stimuli in the face-vignette task and basic facial emotion identification test, was responsible for the ERP characteristics of effortful cognitive processing in participants. N250 amplitudes at the prefrontal site and frontal site in the schizophrenia group were larger than those in the HC group, and N250 latencies in the schizophrenia group were longer than those in the HC group.

This study mainly focused on the investigations of effortful information processing using the effortful cognitive task, and we did not explore the effect of the emotional on individual’s cognition. The BFEIT and FVT only reflected automatic information processing and effortful information processing respectively. In our study, the procedure of the FVT mainly included vignettes describing situational information that was discrepant in affective valence prior to target facial expressions; this resulted in appraisals of emotional attributes reflecting the dominance of either the facial expression or the emotional context. Many studies on effortful cognitive processing have reported that schizophrenia patients present impairments in effortful emotional processing (Rowland et al., 2012; Patrick et al., 2015). Additionally, results of a study suggested that the impaired motivational drive in patients with schizophrenia may be at least partly due to a decreased effort-expenditure for greater rewards (Barch et al., 2014). Consistent with the findings of the above studies, this study confirmed that schizophrenia patients cannot perfectly utilize contextual information for specific story-face pairs, whereas normal controls more commonly made good judgments on the contextual information, which showed that schizophrenia patients present effortful cognitive impairment.

Many studies have indicated that ERP amplitudes, which are evoked by different complexity levels of tasks, reflect electrophysiological measurements of the mental workload (Horat et al., 2016; Shaw et al., 2018), whereas ERP latencies reflect the brain cognitive processing speed for the target stimulus onset (Mcarthur and Bishop, 2002; Tsai et al., 2012). N250 is an ERP component that has been studied in relation to face emotion processing in schizophrenia. Previous studies showed that abnormal N250 is related to the impaired face emotion processing in schizophrenia (Lee et al., 2010; Wynn et al., 2013; McCleery et al., 2015). Additionally, individuals at risk for schizophrenia showed significant impairments in facial affect recognition and reduced amplitudes in the N250 component (Wolfgang et al., 2012), and Positive and Negative Syndrome Scale (PANSS) scores of schizophrenia patients are related to N250 component (Kim et al., 2013). However, studies which investigated the characteristics of ERPs induced by facial emotion recognition displayed that schizophrenia patients did not show N250 abnormalities (Wynn et al., 2013; Yang et al., 2017). Our results showed that N250 amplitudes in schizophrenia patients were larger than those of HCs and N250 latencies in schizophrenia patients were longer than those of HCs, which might suggest that schizophrenia patients experienced an increased mental workload and slowed processing speed due to effortful information processing deficits. The characteristic ERP component N250 triggered by the target stimulus may be a valuable marker for studying schizophrenia.

In our study, the PANSS scores were not correlated with N250 amplitudes and latencies, which might indicate that abnormal N250 are not state dependent in schizophrenia. We assumed that larger N250 amplitude reflected more substantial increases in the cognitive resources allocated to the processing of the effortful cognitive task. Our findings also confirm that neural correlates of effortful cognitive processing deficits in Schizophrenia because of the abnormal N250 components at the prefrontal and frontal sites. Namely, a high load on “prefrontal and frontal site” cognitive processes led to a larger N250 amplitude.

Because no differences were observed between schizophrenia patients and HCs in the ERP component N250, which was triggered by the target stimulus in BFEIT (i.e., the picture of a face in the BFEIT) onset, all participants used a similar amount of cognitive resources. However, our findings for ERP characteristics in the BFEIT and FVT might be due to differences in task demands, i.e., automatic cognition processing requires near zero attention for the task, whereas effortful processes use attentional capacity; the mental workload was higher in the FVT than in the BFEIT.

## Conclusion

Schizophrenia patients present effortful cognitive processing dysfunctions, and effortful cognitive processing deficits show abnormal ERP responses at prefrontal cortex and frontal cortex sites.

## Limitations

There are several limitations in the present study. Firstly, participants’ effortful cognitive processing might be influenced by the working memory since the vignettes and face pictures were presented sequentially. It’s worthwhile to establish better experimental paradigm that can address this problem and further explore the effortful cognitive processing characteristics. Secondly, the schizophrenia patients in this study were clinical stable ones; therefore, whether the results can reflect situations of patients in other disease status remains unclear. Thirdly, because of the small sample sizes, the findings must be considered as preliminary. Further studies with larger sample sizes and with the same ERP parameters are needed to further verify the findings of the study. Finally, because of the deficient spatial resolution of ERPs, further studies with functional magnetic resonance imaging or magnetoencephalography should be conducted to confirm the underlying brain generators, as presented by an abnormality in ERP response, which may further clarify the neural mechanism of effortful cognitive processing deficits in schizophrenia.

## Data Availability Statement

The datasets generated for this study are available on request to the corresponding author.

## Ethics Statement

The studies involving human participants were reviewed and approved by the Ethics Committee on Human Studies, the Affiliated Wuxi Mental Health Center of Nanjing Medical University, Wuxi, Jiangsu Province, China. The patients/participants provided their written informed consent to participate in this study.

## Author Contributions

C-GJ and Z-HZ wrote the manuscript. C-GJ and JW performed the BFEIT and FVT data analysis and statistics. C-GJ, X-HL, Y-LX, and JW oversaw the ERP data/demographic data collection. C-GJ and Z-HZ analyzed the ERP data. Z-HZ was in charge of the design and implementation of the study and contributed to the data interpretation. All authors contributed to the article and approved the submitted version.

## Conflict of Interest

The authors declare that the research was conducted in the absence of any commercial or financial relationships that could be construed as a potential conflict of interest.

## References

[B1] Barch D. M. Treadway M. T. Schoen N. (2014). Effort, anhedonia, and function in schizophrenia: reduced effort allocation predicts a motivation and functional impairment. *J. Abnorm. Psychol.* 123 387–397. 10.1037/a0036299 24886012 PMC4048870

[B2] Bowie C. R. Reichenberg A. Patterson T. L. Heaton R. K. Harvey P. D. (2006). Determinants of real-world functional performance in schizophrenia subjects: correlations with cognition, functional capacity, and symptoms. *Am. J. Psychiatry* 163 418–425. 10.1176/appi.ajp.163.3.418 16513862

[B3] Gong X. Huang Y. X. Wang Y. Luo Y. J. (2011). Revision of the Chinese facial affective picture system. *Chinese Mental Health J.* 25 40–46. 10.3969/j.issn.1000-6729.2011.01.011

[B4] Granholm E. Verney S. P. Perivoliotis D. Miura T. (2007). Effortful cognitive resource allocation and negative symptom severity in chronic schizophrenia. *Schizophr. Bull.* 33 831–842. 10.1093/schbul/sbl040 16956985 PMC2526135

[B5] Hammar A. Ardal G. (2012). Effortful information processing in patients with major depression - a 10-year follow-up study. *Psychiatry Res.* 198 420–423. 10.1016/j.psychres.2011.11.020 22445703

[B6] Horat S. K. Herrmann F. R. Favre G. Terzis J. Debatisse D. Merlo M. C. G. (2016). Assessment of mental workload: a new electrophysiological method based on intra-block averaging of ERP amplitudes. *Neuropsychologia* 82 11–17. 10.1016/j.neuropsychologia.2015.12.013 26724546

[B7] Kim D. W. Lee S. H. Im C. H. (2013). Source activation during facial emotion perception correlates with positive and negative symptoms scores of schizophrenia. *Annu. Int. Conf. IEEE Eng. Med. Biol Soc.* 2013 6325–6328. 10.1109/embc.2013.6611000 24111187

[B8] Lee S. H. Kim E. Y. Kim S. Bae S. M. (2010). Event-related potential patterns and gender effects underlying facial affect processing in schizophrenia patients. *Neurosci. Res.* 67 172–180. 10.1016/j.neures.2010.03.001 20214929

[B9] MacKenzie N. E. Kowalchuk C. Agarwal S. M. Costa-Dookhan K. A. Caravaggio F. Gerretsen P. (2018). Antipsychotics, metabolic adverse effects, and cognitive function in schizophrenia. *Front. Psychiatry* 9:622. 10.3389/fpsyt.2018.00622 30568606 PMC6290646

[B10] Mcarthur G. Bishop D. J. N. (2002). Event-related potentials reflect individual differences in age-invariant auditory skills. *Neuroreport* 13 1079–1082. 10.1097/00001756-200206120-00021 12060813

[B11] McCarthy J. M. Treadway M. T. Bennett M. E. Blanchard J. J. (2016). Inefficient effort allocation and negative symptoms in individuals with schizophrenia. *Schizophr. Res.* 170 278–284. 10.1016/j.schres.2015.12.017 26763628 PMC4740196

[B12] McCleery A. Lee J. Joshi A. Wynn J. K. Hellemann G. S. Green M. F. (2015). Meta-analysis of face processing event-related potentials in schizophrenia. *Biol. Psychiatry* 77 116–126. 10.1016/j.biopsych.2014.04.015 24923618

[B13] Monaghan C. K. Brickman S. Huynh P. Öngür D. Hall M. H. (2019). A longitudinal study of event related potentials and correlations with psychosocial functioning and clinical features in first episode psychosis patients. *Int. J. Psychophysiol.* 145 48–56. 10.1016/j.ijpsycho.2019.05.007 31108121 PMC6988566

[B14] Ng-Mak D. Messali A. Huang A. Wang L. Loebel A. (2019). Hospitalization risk in patients with schizophrenia treated with dose-equivalent antipsychotics. *Am. J. Manag. Care* 25 S279–S286. 31365818

[B15] Ochsner K. N. Gross J. J. (2005). The cognitive control of emotion. *Trends Cogn. Sci.* 9 242–249. 10.1016/j.tics.2005.03.010 15866151

[B16] Patrick R. E. Rastogi A. Christensen B. K. (2015). Effortful versus automatic emotional processing in schizophrenia: insights from a face-vignette task. *Cogn. Emot.* 29 767–783. 10.1080/02699931.2014.935297 25034611

[B17] Phillips M. L. Drevets W. C. Rauch S. L. Lane R. (2003). Neurobiology of emotion perception II: implications for major psychiatric disorders. *Biol. Psychiatry* 54 515–528. 10.1016/s0006-3223(03)00171-9 12946880

[B18] Rodriguez-Larios J. Faber P. Achermann P. Tei S. Alaerts K. J. (2020). From thoughtless awareness to effortful cognition: alpha - theta cross-frequency dynamics in experienced meditators during meditation, rest and arithmetic. *Sci. Rep.* 10:5419. 32214173 10.1038/s41598-020-62392-2 PMC7096392

[B19] Rowland J. E. Hamilton M. K. Vella N. Lino B. J. Mitchell P. B. Green M. J. (2012). Adaptive associations between social cognition and emotion regulation are absent in schizophrenia and bipolar disorder. *Front. Psychol.* 3:607. 10.3389/fpsyg.2012.00607 23423878 PMC3573888

[B20] Rund B. R. Landrø N. I. (1990). Information processing: a new model for understanding cognitive disturbances in psychiatric patients. *Acta Psychiatr. Scand.* 81 305–316. 10.1111/j.1600-0447.1990.tb05455.x 2188480

[B21] Shaw E. P. Rietschel J. C. Hendershot B. D. Pruziner A. L. Miller M. W. Hatfield B. D. (2018). Measurement of attentional reserve and mental effort for cognitive workload assessment under various task demands during dual-task walking. *Biol. Psychol.* 134 39–51. 10.1016/j.biopsycho.2018.01.009 29378284

[B22] Stern P. (2012). Countering impaired cognition. *Science* 338:444. 10.1126/science.338.6106.444-c

[B23] Tancer M. E. Brown T. M. Evans D. L. Ekstrom D. Golden R. N. (1990). Impaired effortful cognition in depression. *Psychiatry Res.* 31 161–168. 10.1016/0165-1781(90)90118-O 2326395

[B24] Tomasello M. Call J. (2011). Methodological challenges in the study of primate cognition. *Science* 334 1227–1228. 10.1126/science.1213443 22144614

[B25] Tsai M. L. Hung K. L. Lu H. P. (2012). Auditory event-related potentials in children with attention deficit hyperactivity disorder. *Pediatr Neonatol.* 53 118–124. 10.1016/j.pedneo.2012.01.009 22503259

[B26] Wolfgang W. Brinkmeyer J. Stroth S. Streit M. Bechdolf A. Ruhrmann S. (2012). Neurophysiological correlates of impaired facial affect recognition in individuals at risk for schizophrenia. *Schizophr Bull.* 38 1021–1029. 10.1093/schbul/sbr013 21402721 PMC3446235

[B27] Wynn J. K. Jahshan C. Altshuler L. L. Glahn D. C. Green M. F. (2013). Event-related potential examination of facial affect processing in bipolar disorder and schizophrenia. *Psychol. Med.* 43 109–117. 10.1017/s0033291712001006 22583955 PMC3959981

[B28] Yang C. Zhang T. Li Z. Heeramun-Aubeeluck A. Liu N. Huang N. (2017). Changes in event-related potentials in patients with first-episode schizophrenia and their siblings. *BMC Psychiatry* 17:20. 10.1186/s12888-016-1189-7 28095817 PMC5240372

[B29] Yank G. R. Bentley K. J. Hargrove D. S. (1993). The vulnerability-stress model of schizophrenia: advances in psychosocial treatment. *Am. J. Orthopsychiatry* 63 55–69. 10.1037/h0079401 8427312

